# Systems Thinking in an era of climate change: Does cognitive neuroscience hold the key to improving environmental decision making? A perspective on Climate-Smart Agriculture

**DOI:** 10.3389/fnint.2023.1145744

**Published:** 2023-04-27

**Authors:** Baqir Lalani, Steven Gray, Tora Mitra-Ganguli

**Affiliations:** ^1^Natural Resources Institute, University of Greenwich, Chatham Maritime, United Kingdom; ^2^Department of Community Sustainability, Michigan State University, East Lansing, MI, United States; ^3^Independent Researcher, West Kirby, United Kingdom

**Keywords:** Global South, Systems Thinking (ST), climate change, mobile data collection, Climate Smart Agriculture (CSA)

## Abstract

Systems Thinking (ST) can be defined as a mental construct that recognises patterns and connections in a particular complex system to make the “best decision” possible. In the field of sustainable agriculture and climate change, higher degrees of ST are assumed to be associated with more successful adaptation strategies under changing conditions, and “better” environmental decision making in a number of environmental and cultural settings. Future climate change scenarios highlight the negative effects on agricultural productivity worldwide, particularly in low-income countries (LICs) situated in the Global South. Alongside this, current measures of ST are limited by their reliance on recall, and are prone to possible measurement errors. Using Climate-Smart Agriculture (CSA), as an example case study, in this article we explore: (i) ST from a social science perspective; (ii) cognitive neuroscience tools that could be used to explore ST abilities in the context of LICs; (iii) an exploration of the possible correlates of systems thinking: observational learning, prospective thinking/memory and the theory of planned behaviour and (iv) a proposed theory of change highlighting the integration of social science frameworks and a cognitive neuroscience perspective. We find, recent advancements in the field of cognitive neuroscience such as Near-Infrared Spectroscopy (NIRS) provide exciting potential to explore previously hidden forms of cognition, especially in a low-income country/field setting; improving our understanding of environmental decision-making and the ability to more accurately test more complex hypotheses where access to laboratory studies is severely limited. We highlight that ST may correlate with other key aspects involved in environmental decision-making and posit motivating farmers *via* specific brain networks would: (a) enhance understanding of CSA practices (e.g., *via* the frontoparietal network extending from the dorsolateral prefrontal cortex (DLPFC) to the parietal cortex (PC) a control hub involved in ST and observational learning) such as tailoring training towards developing improved ST abilities among farmers and involving observational learning more explicitly and (b) motivate farmers to use such practices [e.g., *via* the network between the DLPFC and nucleus accumbens (NAc)] which mediates reward processing and motivation by focussing on a reward/emotion to engage farmers. Finally, our proposed interdisciplinary theory of change can be used as a starting point to encourage discussion and guide future research in this space.

## 1. Introduction

Systems Thinking (ST) can be defined as a mental construct that recognises patterns and connections in a particular system to make the “best decision” possible given a particular goal. In a number of environmental fields including sustainable agriculture and fisheries management, higher degrees of ST are thought to be associated with “better” (e.g., optimal given preferred outcomes and understood constraints environmental decision making in a variety of country settings (e.g., Gray, 2018; Lalani et al., 2021; Aminpour et al., 2022). For example, recent studies in a North American setting have shown that higher degrees of ST correlate with the use of conservation practices, including pest management practices (e.g., Bardenhagen et al., 2020), holistic management/agricultural practices (Mann et al., 2019) and cover crops (Church et al., 2020). Higher degrees of ST have also been associated with the use of Conservation Agriculture (CA) in Mozambique (e.g., Lalani et al., 2021) and more recently with sustainable groundwater management in India (Sanga and Koli, 2023). Social science methods have often measured degrees of “systems thinking” by exploring the qualitative and quantitative attributes of individual mental models often through analysing concepts or cognitive maps. Methods used to elucidate mental models through cognitive maps such as Fuzzy Cognitive Mapping (FCM), rely upon participants to represent their thinking process. Yet defining and measuring “systems thinking” remains challenging (Gray, 2018). Thus, tacit knowledge, and subconscious cognitions remain inaccessible, even though they may play important roles in systems thinking and/or environmental decision making especially since such decisions rely on: (1) understanding a system’s composition and (2) incorporating and adapting to new changing conditions as they happen to make both short term and long term strategies for human behaviour. Additionally, complexities have been found in other areas of sustainable behaviour which rely on self-reports which are prone to biases. Leeuwis et al. (2022) suggest that neuroscience tools can provide an additional implicit measurement, for instance, when the verbalised attitudes/intention reported may not be consistent with actual behaviour. Moreover, studies that look at neuroscience and sustainability are scarce and fragmented (Leeuwis et al., 2022). Furthermore, digital mobile technologies whilst not novel in the field of cognitive neuroscience and/or cognitive psychology have mostly been utilised in highly controlled settings and in higher-income countries (Bhavnani et al., 2022). Sawe (2019) has further argued that the benefits of neuroscience tools in the area of environmental policy research provide a number of benefits including improving our understanding of how decision making differs among individuals; the specific behavioural nudges that can have an influence on decision making and the ability to make population level inferences by looking at what types of decision making processes are predictive of national behaviour. All of these may overlap and interact depending on the scale/population of interest (ibid). Furthermore, engaging lower-income countries in such research will be important given the majority of the world resides in lower-income countries and these are the populations most likely to benefit from such research as such “thinking” is culturally embedded (Valdes-Sosa et al., 2021).

### 1.1. Other examples of social science frameworks used to explore environmental decision making^1^

A number of social science frameworks and methods have been used to explore environmental decision making (including farmers’ decision making) more broadly. Some examples include Multi-Criteria Decision Analysis (MCDA) (e.g., Kiker et al., 2005) and the utilisation of Agent-Based Modelling (ABM) such as Zolfagharipoor and Ahmadi (2021) who employ the ABM approach to simulate a local groundwater market in central Iran and incorporate the theory of planned behaviour to explore the agents’ intention of participating in the market. Similarly, Streefkerk et al. (2023) also used ABM and coupled a spatially distributed hydrological model to a human behavior-centered ABM and found agropastoralists in Kenya respond differently to drought due to differences in perceptions of their environment. Benhangi et al. (2020) recently employed an interesting methodological framework to assess the “learning capacity” (incorporating the learning process and learning outcomes) of water users in Iran and found that water users’ responses were associated with factors such as social memory which negatively impacted water use. Other authors have developed a socio-cognitive conceptual framework that explicitly considers feedback from ecosystems to land use systems and how changes in ecosystems services then reflect in land management decisions. The authors’ found farmers’ behaviours were not always synonymous with their attitudes towards ecosystem services (i.e., their decisions on changes in ecosystem services were not reflective of their underlying beliefs towards ecosystem services) and other factors including topographic constraints or farmer individual and household characteristics also played a part in land-management decisions (Lamarque et al., 2014). Finally in a review article, seeking to understand how people make decisions and analyse social-ecological systems, Binder et al. (2013) analysed 10 established frameworks for analysing social-ecological systems and found that there are three types; those exploring the social impact on the environment, others focussed on the environmental impact on social domains and those that incorporate both social and environmental impacts with feedback loops.

But how can current social science frameworks and advancements in cognitive neuroscience be used to better understand culturally embedded knowledge or ways of thinking, ST, and human decision making, especially since climate change and human thinking and responses are critical for all human societies?

We posit that in addition to traditional social science methodologies of measuring ST, integrating cognitive neuroscience approaches that measure brain activity, can detect neural correlates^2^ of these otherwise inaccessible cognitive patterns. By pairing cognitive neuroscience methods with social or psychological measures of systems thinking, we can not only access hidden forms of cognition, but we can also begin to outline the neural mechanisms and corresponding psychological processes involved in improved environmental decision making. The following uses Climate Smart Agriculture (CSA) as a case study with the backdrop of the importance of applying these tools/approaches to low-income country (LICs) settings.^3^

### 1.2. A case study of environmental decision making: the case of climate smart agriculture

Agricultural production contributes substantially to climate change: yearly greenhouse gas (GHG) emissions from agriculture account for 11% of total anthropogenic GHG emissions, not including land use change from natural vegetation/forests to agriculture (Poeplau and Don, 2015; De Pinto et al., 2020). The significant role that agriculture plays in contributing to climate change has increased the importance of Climate Smart Agriculture (CSA) both from the potential of contributing to mitigation and also more importantly to climate change adaptability. CSA is an approach based on three main objectives: (i) sustainably increasing agricultural productivity and incomes; (ii) adaptation and building of resilience to climate change; and (iii) reducing and/or removing greenhouse gas emissions, where possible (FAO, 2022a). A CSA practice is considered to be context-specific and dependent on a range of factors (e.g., local, socio-economic, and environmental factors) and implemented at the field level (FAO, 2022a).

CSA practices have been associated with improvements in natural resource sustainability (e.g., soil and land) and preservation of vital ecosystems which contribute to enhancing resilience and climate change induced vulnerabilities both at the farm/household level and wider landscape level (Saran et al., 2022). Future climate change scenarios have increasingly highlighted the negative effects on agricultural productivity worldwide (e.g., Nelson et al., 2014) and this is likely to be especially acute in low-income countries (LICs), particularly for those situated in the Global South (e.g., Morton, 2007).

### 1.3. CSA in Africa

Two-thirds of the world’s poorest people reside in rural areas (76% are located in Africa) and are primarily engaged in agriculture (World Bank, 2014; IFAD, 2020). Although Africa has had the largest annual rate of net forest loss (3.9 million hectares) over the period 2010–2020 (FAO, 2020) and this has steadily increased in recent decades; Africa is still the smallest contributor to global greenhouse gas emissions though the most vulnerable to the impacts of climate change (Gonzalez-Sanchez et al., 2019). Some authors have suggested there exists limited scope for carbon sequestration *via* certain CSA practices such as crop residue retention due to the “sink saturation effect” i.e., a point being reached when no net carbon sequestration takes place beyond this; the authors do point out that improving the organic matter in soils is still desirable given changing conditions (e.g., Berthelin et al., 2022). Moreover, recent studies at a regional level in Africa (Gonzalez-Sanchez et al., 2019) and modelling at a global scale have suggested that these practices can increase food production for millions of people and reduce GHG emissions (De Pinto et al., 2020). For example, CA has been associated with an increase in productivity, improvements in household income, and enhanced food security at the household-level in Mozambique (e.g., Nkala et al., 2011; Lalani et al., 2021). Others have found that CA usage in Zambia, for instance, has substantially increased maize production and reduced household poverty (Abdulai, 2016)^4^. However, farmers must identify what is considered “climate-smart” in their own contexts (e.g., biophysical, socio-economic, etc.) (De Pinto et al., 2020). Whilst there have been many successes (e.g., Kassam et al., 2017), CSA practices are often perceived as “knowledge-intensive” and notwithstanding other constraints it has been suggested this can deter farmers from using such practices (e.g., De Pinto et al., 2020). In a number of LICs (including in Sub-Saharan Africa), the use/local adaptation of practices remains low^5^ (Makate, 2019). Practices such as crop burning and tillage are widely used which have led to widespread soil degradation further limiting the potential for agriculture production (Rockström et al., 2009). It is important to note, however, that elements of “modern” agriculture and the application of science and technology have been historically linked to colonial structures in Africa (Moyo, 2010). For example, the focus on monocultures and subsequent investment in the processing of sugarcane and other crops (e.g., tobacco) in Malawi stemmed from white settlers in the 1800s (Buchanan, 1885; Woods, 1993; Moyo, 2010). Moreover, settlers were amazed to find local people cultivating crops such as maize and beans in mixtures (e.g., intercropping which is considered to be among the oldest indigenous agriculture production techniques in tropical Africa) as well as the practice of minimum tillage as local farmers tilled the land at a very shallow depth (less than 25 cm deep) which was described as a “mere scratching of the soil surface” (Buchanan, 1885 cited in Moyo, 2010; Rogé et al., 2016) It has thus been argued that land degradation is in part attributed to a legacy of colonial policies which discouraged these indigenous practices (Rogé et al., 2016). Thus, Moyo et al. (2022) have recently advocated for the co-creation of knowledge which includes farmers’ indigenous knowledge (local knowledge) and scientific knowledge thereby leading to more holistic knowledge^6^. Our focal point is thus SSA where both culture (ways of thinking) and environmental conditions (regional and local) are immensely diverse; farmers’ indigenous knowledge has historically often been discarded (e.g., Kerr et al., 2022; Moyo et al., 2022) and demand for food and nutrition security/climate change adaptation remains constant if not increasing (FAO, 2021). Recently, neuroscience researchers have also called on the neuroscience community to conduct more work globally on environmental conservation including the use of no-tillage (i.e., forms part of CSA practices) (Keifer and Summers, 2021).

But do farmers that use CSA practices have individual thinking patterns that are unique from those that do not (e.g., increased or decreased ST or not) and how do we know what types of thinking lead to better human adaptations to a changing climate?

This perspective essay outlines a research agenda to explore systems thinking from both a social science perspective and a cognitive neuroscience lens in order to help elucidate key mechanisms of decision making with respect to CSA practices. The article is structured as follows: (i) ST from a social science perspective; (ii) cognitive neuroscience tools that could be used to explore ST abilities in the context of LICs; (iii) an exploration of the possible correlates of systems thinking: observational learning, prospective thinking/memory and the theory of planned behaviour and (iv) a proposed theory of change highlighting the integration of social science frameworks and a cognitive neuroscience perspective that can be used to enhance our understanding of ST and farmers’ decision making.

## 2. Systems thinking—a social science perspective

Measures of ST have indicated decision-makers that show more evidence of ST/indicators correlate with more desirable human and environmental outcomes (see Aminpour et al., 2022) given competing outcomes and depending on what the decision-maker needs wants to optimise. However most of this research has been limited to social science disciplines. While ST has been a popular approach for decades to understand “better” and value-laden decision making (Stave and Hopper, 2007; Skaza and Krystyna, 2009), there remain significant gaps in understanding how ST is promoted and how to assess and measure ST understanding. The popularity of promoting ST across disciplines is based on two major benefits. First, ST relies on the notion that if decision-makers, formally or informally, can develop skill sets that allow them to think deeply (and demonstrate that through cognitive mapping empirical evidence) about the complex dynamics of systems, they are better prepared to predict a system’s behaviour, and engineer solutions that lead to more favourable outcomes (see identifying “leverage points” discussed in Meadows, 2008). Additionally, since ST is a highly generic, synthetic, and generalizable construct, it can also be a useful way for decision-makers to integrate and synthesise knowledge across domains (Arnold and Wade, 2015). Such systemic thinking generates habits of mind (Kay and Foster, 1999; Steinkuehler and Duncan, 2008) that are useful frameworks for reasoning about and abstracting over a range of systems that underlie personal or global problems (Tabacaru et al., 2009). For example, Sterling et al. (2010) have argued that a systems view of the interacting biophysical and cultural systems at the core of biological diversity can result in more effective conservation targets and strategies.

### 2.1. The importance of understanding individual mental models

To understand individual farmers’ perceptions, research has traditionally focused on understanding and measuring their “mental models” as they relate to CSA and behaviours. The notion of mental models, which was first introduced by Craik (1943), has been widely used to study how individuals and groups understand the world and make decisions within it (see review by Jones et al., 2011). These internal models are often elicited and represented through concept or cognitive mapping. A cognitive map can be thought of as a graphical map that reflects mental processing, which is comprised of collected information and a series of cognitive abstractions by which individuals filter, code, store, refine and recall information about physical phenomena and experiences into an external representation (Vanwindekens et al., 2013; Vuillot et al., 2016; Levy et al., 2018). Therefore, understanding variation in farmer mental models, and indeed in some cases how consistent these perceptions align with measurements of external “reality”, can shed light on human decision making and subsequent behavioural intentions and behaviours (Halbrendt et al., 2014).

### 2.2. Concept mapping to represent mental models

Concept mapping is often used to externally represent individual mental models and as an additional tool to explore dimensions of ST. For example, knowledge of a specific topic is represented graphically with directional lines used to illustrate relationships between concepts (Novak and Cañas, 2006). It has also been used in prior research exploring students’ ST with respect to sustainability issues (Brandstädter et al., 2012). Concept generation is a process of first-order thinking involving memorization and knowledge combination, and also higher-order thinking involving memorizing, reasoning, relational thinking, and knowledge organization (Zvacek et al., 2012; Taura and Nagai, 2013).

### 2.3. Using Fuzzy cognitive maps to represent mental models

One recent and semi-quantitative way to measure individual mental models has come through Fuzzy Cognitive Mapping (FCM). FCM has been used in many contexts ranging from fisheries management to agricultural development to generate graphical models of complex systems that are useful for decision making, illuminate the core presumptions of local stakeholders, structure complex problems for scenario development, and understand degrees of ST (e.g., Halbrendt et al., 2014; Lalani et al., 2021). FCM has become popular because it takes a bottom-up approach and can incorporate a range of individual, community-level, and expert knowledge into an accessible and standardized format to better understand individual mental model variation among communities or stakeholder groups but also elucidate more the more “community-level” understanding that to some extent highlights societal understanding and their associated behaviours (see Aminpour et al., 2022). FCMs are semi-quantitative instantiations of graph theory, the structure between state space variables can be represented mathematically. These structural measures are generated by converting cognitive maps into an adjacency matrix filled with positive or negative values that define relationships between variables on a scale between +1 and −1. Representing the structural relationships of these concepts in a matrix allows each variable to be categorized in one of three ways: (1) as a driving variable, i.e., forcing component; (2) receiving variable, i.e., impacted component; or (3) an ordinary variable, i.e., intermediate component (Nayaki et al., 2014). A variable’s relative importance for the system can be determined by the strength of its incoming and outgoing edges using centrality measurements common to network analyses (see Özesmi and Özesmi, 2004). FCMs can also be characterized by a range of other quantitative metrics, including density, which allows models to be compared with each other based on their overall structure (see Gray et al., 2015 for a review of structural metrics). Importantly, FCMs can run “what-if” scenarios (Kosko, 1986; Özesmi and Özesmi, 2004). That is, FCM computation can show the relative changes in the state of the system’s components given a particular input or combination of inputs (i.e., a forced manipulation in the state of the system, also known as system “activation”). When one component is activated (i.e., sends a signal), it triggers a cascade of changes to other system components based on how they are connected and in this way represents the dynamics of a personal scenario in an individual’s mental model. This process continues in several iterations until the initial signal has passed through the entire FCM and all components reach a steady state. By comparing the system state at the beginning with that at the end of the process, we can assess the direction and strength of impact that the change has had on all other components. Such FCM simulations provide the toolset for a dynamic analysis of mental models and have been used by many researchers to represent belief-based predictions (e.g., Jones et al., 2011; Halbrendt et al., 2014; Stier et al., 2017; Cholewicki et al., 2019). For more information about the scenario analysis and equations see Özesmi and Özesmi (2004) and Aminpour et al. (2020).

### 2.4. Measuring degrees of systems thinking using network analysis

Systems thinking is an important skill that helps humans understand and manage complex systems (Senge and Sterman, 1992) and because of FCMs semi-quantitative and dynamic analytical capabilities, research has recently begun to define network-based metrics with degrees of ST. In particular, the ability to define components and understand the dynamics of a system in a systematic way can improve farmers’ engagement with sustainability issues, because these are always complex with intertwined social, environmental, and economic aspects (Aminpour et al., 2022). Farmers who use higher degrees of systems thinking can better understand the complex dynamics of a system: they are more likely to better predict a system’s behaviour, identify leverage points (Meadows, 2008), and evaluate the trade-offs between different decisions made within the system. In addition, Levy et al. (2018) have shown that the degree of “systems thinking” can be measured using network analysis of mental models that represent perceived causal structures between system components. As such, network metrics that measure the degree of complexity, non-linearity, non-hierarchical causation, cyclic (closed loop) interdependence, and feedback representation may exemplify higher levels of systems thinking. So-called “micro-motifs” allow for the clustering of cognitive maps on a spectrum to indicate the degree of systems thinking in decision making mental models.

## 3. Measuring systems thinking through the use of mobile tools in LICs and a cognitive neuroscience lens

While FCM and related network approaches have made some strides in measuring systems thinking, they are limited by their reliance upon participants’ meta-cognition, recall, honesty, and ability to articulate their thoughts and possible measurement errors given the complexity of the task. This leaves early, subconscious, tacit, and socially-undesirable patterns of thinking inaccessible, even though those may play important roles in environmental decision making. In contrast, neuroscience measures of brain activity can access these kinds of cognition by observing their neural correlates by pairing the network measures from concept mapping with brain activity (e.g., Hu et al., 2019).

**Figure 1:**
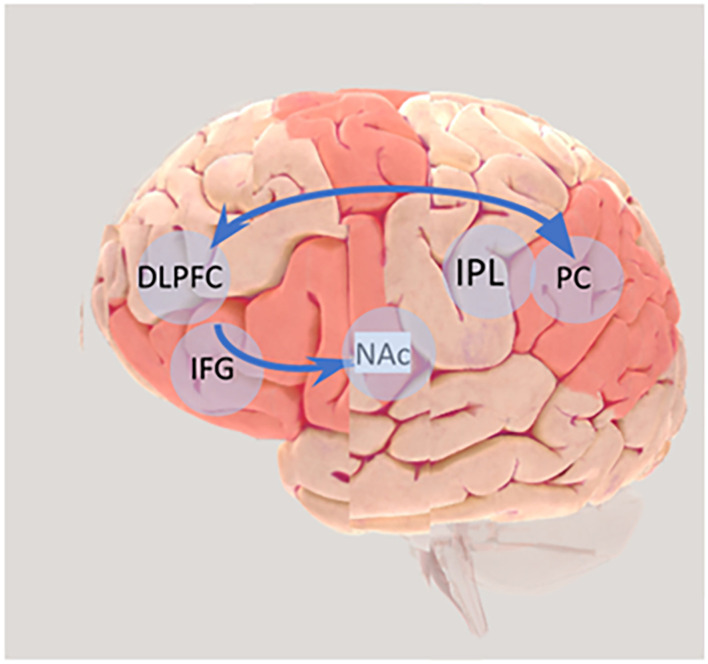
The frontoparietal network, indicated by the arrow, extending from the dorsolateral frontal (DLPFC) cortex to the parietal cortex (PC) is a control hub involved in Systems Thinking (ST), observational learning. This region also overlaps with the putative mirror neuron system extending from the inferior frontal gyrus (IFG) to the inferior parietal lobule (IPL). The network between the DLPFC and nucleus accumbens (NAc) mediates reward processing and motivation which is important for observational learning. DLPFC is also linked with pro-environmental behaviour. Source: Image of Brain produced using 3D Brain (brainfacts.org).

Systems thinking relies on efficiency, effectiveness, and reliability (Grohs et al., 2018) of a complex neuroarchitecture. The neural activity that governs our everyday lives involves an intricate coordination of many processes that can be attributed to a variety of brain regions. At best, the numerous dynamic networks underpinning systems thinking can be understood using a systems-level approach such as neuroimaging (Hu and Shealy, 2018; Hu et al., 2019) which enables the collection of objective physiological data during cognitive activity. Hu et al. (2019) used functional near-infrared spectroscopy (fNIRS) to measure and compare BOLD response among engineering students during concept generation and concept listing exercises, to measure systems thinking, for grand challenges to sustainability. The authors showed that engineering students generated significantly more concepts when using concept maps than making linear lists. During tasks of mapping and listing concepts, the BOLD response which is a measure of cognitive activation, was significantly different in two brain regions: the dorsolateral prefrontal cortex (DLPFC) and parietal cortex (PC) (Figure 1). This is a significant finding because both the DLPFC and PC are brain regions known to be involved in higher order executive functions, adaptive thinking (Bembich et al., 2014), and sequence processing (Köhler et al., 1995), all key components of concept mapping.

To measure systems thinking in “field” settings in LICs, the field neuroimaging protocol needs to outline considerations for travelling with and setting up a portable neuroimaging laboratory in low-resource contexts.

### 3.1. What are the appropriate tools that can be used in a LIC setting?

When considering the appropriate neuroimaging tool, it is worth bearing in mind that electroencephalogram (EEG) provides temporal resolution in the milliseconds range while functional magnetic resonance imaging (fMRI) provides a high level of spatial resolution. Recent methodologies such as functional Near Infrared Spectroscopy (fNIRS) provide better temporal resolution than fMRI and better spatial resolution than EEG (Lloyd-Fox et al., 2010). Researchers use fNIRS to study experimental tasks related to thinking (Pike et al., 2014), decision making (Cazzell et al., 2012), and problem-solving (Leff et al., 2009) because it is more ambulatory compared to EEG and allows for the flexibility to study human cognition in “real life” settings compared to fMRI (Irani et al., 2007). Both fNIRS and fMRI measure changes in oxygenated blood, or oxygenated haemoglobin, and deoxygenated haemoglobin to give a readout of brain activity. fNIRS neuroimaging is well-suited for field research (Baker et al., 2017). A key advantage of fNIRS is its portability (i.e., some systems may fit in a suitcase), ease of use, and the fNIRS system also tolerates movement well compared to fMRI. fNIRS have superior temporal resolution to fMRI and also has good spatial resolution; the fNIRS’ depth of recording in the human cortex is less than fMRI, measuring about 3–4 cm in depth, which is well-suited for studying cortical functions (Jasińska and Guei, 2018). A limitation of using fNIRS is that the spatial resolution is limited compared to fMRI and therefore considered less appropriate when deeper brain structures (such as the nucleus accumbens) are of primary interest (Kopton and Kenning, 2014). This could be a limitation of using the technology, but in the absence of better portable technology fNIRS could offer a reasonable solution. Mobile EEG tools are also available for recording brain activity and have field recording potentials. However, these are more applicable to consumer applications. While recent research has demonstrated the accessibility, feasibility, and usability of Electroencephalography (EEG; e.g., EMOTIV +) in a rural area (predominantly agricultural area) of India. Similar research using EEG has been conducted in Malawi, The Gambia, and Bangladesh (Bhavnani et al., 2022), and further work is required to establish mobile EEG methodologies for neurodevelopmental research (Lau-Zhu et al., 2019). Compared to more traditional research-grade high-density EEG systems, mobile EEG has been used in a limited number of research studies and has better applications in sports, neurofeedback, and motor rehabilitation. For this type of research (e.g., exploration of neural correlates of ST) neuroscience experts from the University of Geneva have recommended using Near-Infrared Spectroscopy (NIRS) (e.g., https://neurolite.ch/en/products/nirs/portalite-mkii; University of Geneva, Personal Communication). Advantages include better spatial coverage, rapid onboard data collection, and the ability for a non-specialist with relatively limited training to gather data (ibid). Whilst it has been noted that EEG and fNIRS have excluded participation among participants due to hair structure, skin pigmentation, etc. (e.g., Green et al., 2022; Webb et al., 2022), these alternative designs allow for the ability to engage a wide variety of participants irrespective of skin pigmentation^7^ (University of Geneva, Personal Communication).

## 4. Correlates of systems thinking: observational learning, prospective thinking/memory and the theory of planned behaviour?

We have highlighted in the previous section how such methodologies may be incorporated in an “in-the-field” setting particularly in an LIC context. The task ahead is to then explore to what extent these neural correlates of ST are associated with other aspects involved in environmental decision making. The following makes the case for including observational learning, prospective thinking/memory, and constructs that form part of the theory of planned behaviour that have played important roles in our understanding of environmental decision making including CSA practices/other pro-environmental behaviours in a wide variety of country settings (e.g., Kondylis et al., 2016; Lalani et al., 2016; Maertens et al., 2020; Doell et al., 2021).

### 4.1. Observational learning

Observational learning occurs through the observation of others even when this may happen in the context of other activities (Fryling et al., 2011). It requires observing the actions of others which is also known to vicariously recruit brain regions traditionally associated with action execution (Rizzolatti and Craighero, 2004; Gazzola and Keysers, 2009; Caspers et al., 2010). Several studies have reported that the fronto-parietal human mirror neuron system (hMNS) is strongly recruited while observing actions during the learning of new motor patterns through imitation of other’s actions (Buccino et al., 2004; Vogt et al., 2007; Fabbri-Destro and Rizzolatti, 2008; Cross et al., 2009). The same hMNS is also activated when participants simply view the actions of others without needing to replicate them, or when they simply execute these actions (Gazzola and Keysers, 2009).

The hMNS was found to be strongly activated while participants were observing others’ actions during the acquisition of motor patterns (Caspers et al., 2010). Traditionally, cognitive neuroscience has therefore focussed on the hMNS (Ramsey et al., 2021).

More recently, this has extended beyond the hMNS and involved the extended motor network. Whilst there are a number of types of observational learning (see Ramsey et al., 2021 for a comprehensive review) we refer to the subtype of observational learning (observational motor learning) that requires: (i) an action being observed; and (ii) an enduring change to motor performance must occur (Ramsey et al., 2021). Two types of tasks are involved, namely: (i) sequence learning (e.g., learning to dance or ride a bike) usually measured by serial reaction time, and (ii) motor adaptation (concerned with maintaining consistent performance in light of bodily/environmental changes) studying using visuomotor adaptation tasks (Ramsey et al., 2021).

Calvo-Merino et al. (2006) showed that mirror neuron development relates to the previous motor experience of performing that action; importantly highlighting there are differences by gender. In this study, expert dancers were shown videos of ballet moves that were familiar to both genders. Interestingly, when dancers viewed moves from their “own motor repertoire” (i.e., in this case gender) higher premotor, parietal, and cerebellar activity was found (Calvo-Merino et al., 2006). It is also argued that sensorimotor experience enables mirror neurons to be created by the experience of observing and practicing the action (Heyes, 2009). Interesting parallels can be made with the knowledge and use of sustainable land management techniques. Kondylis et al. (2016) found in communities that were randomly selected to have a trained female extension officer in Mozambique (and encouraged to train other women) that higher levels of knowledge and adoption of pit planting (CSA practice) were found among women farmers.

### 4.2. Current observational learning research methods

Three main types of task design are currently used in observational learning research (Kang et al., 2021). Monfardini et al. (2008) employed a visuomotor learning task design where participants were tasked to watch an actor making motor responses according to the stimulus presentation with post-response feedback (i.e., a binary response regarding whether the actor made the right choice where their reaction times were also recorded). One advantage of this design is that it allows the detection of brain activity when participants retrieve rules (Kang et al., 2021). Monfardini et al. (2013) later built on this design by introducing the learning by observation task (LeO) whereby participants (whilst being scanned by fMRI) were asked to learn stimulus-response associations by watching a video demonstration of an expert performing the correct visuomotor associations which enabled the identification and comparison of brain networks “mediating processing of errors and successes during individual and observational learning” (Monfardini et al., 2013). Burke et al. (2010) employed an observational learning task “two-armed bandit problem” where participants had to make a choice based on two abstract stimuli to either gain a stochastic reward or avoid a stochastic punishment. Of the two stimuli one provided a consistently good outcome (reward or absence of punishment 80% of the time) and the other a consistently bad outcome (punishment or absence of a reward 20% of the time) whilst being scanned by fMRI.

### 4.3. A systems approach to understanding observational learning

Various researchers have suggested that direct simulation of observed social events through mirror-like mechanisms are at the heart of this experiential understanding of others by activation of matching neural substrates in the observer through which the action can be understood (Rizzolatti et al., 2001; Gallese, 2003; Wicker et al., 2003; Goldman and Sripada, 2005; Keysers and Gazzola, 2006). While some researchers focus on the role of motor areas in social cognition (e.g., motor theory of social cognition, Jacob and Jeannerod, 2005), others describe a more embodied simulation that involves a linkage between the first and third person experiences of actions, sensations, and emotions (Keysers and Gazzola, 2006). Although there is no doubt that one can understand others’ emotions *via* inferential mental processes (as during the observation of emotions), there is clear evidence that brain structures involved in the integration and control of emotions, like the insula and the anterior cingulate, respond both when one feels an emotion (e.g., pain or disgust) owing to natural stimuli, or when one observes that emotion in others (Carr et al., 2003; Gallese, 2003; Wicker et al., 2003; Singer et al., 2004). This is a relevant mechanism which could be hypothesised to allow a direct first-person understanding of others’ emotions, especially in the context of positive emotions (e.g., Doell et al., 2021). Doell et al. (2021) showed that observational learning played a key role in commitment to pro-environmental behaviours. More specifically, those with higher levels of positive trait affect (those that tend to experience positive emotions with respect to positive environmental outcomes) were found to commit more pro-environmental behaviour and achieve greater shifts in positive state. These shifts occurred for pro-environmental behaviour that was committed by the individual and for those that were learned from others (observed).

Thus, it has been argued that the process of learning by observation is mediated by brain regions encompassing the dorsal fronto-parietal, the fronto-striatal, and the cerebellar networks. It partly exploits the same neural system mediating individual learning, visuomotor transformations, and the control of goal-direct attention (Monfardini et al., 2013). As a flexible hub of cognitive control, the frontoparietal network carries information about the items stored in working memory and governs the cascade of attentional processes that underlie complex cognitive functions and fluid intelligence (Duncan, 2010, 2013; Stoewer et al., 2010). Functional connectivity between the frontoparietal network and the nucleus accumbens which is involved in reward processing and motivation may also be involved in learning by observation. Hence, we hypothesize that ST leverages the same cognitive flexibility of the prefrontal cortex, involving either the dorsolateral prefrontal cortex (DLPFC) or the parietal cortex, that drives observational learning (Kang et al., 2021) (Figure 1).

For example, Maertens et al. (2020) found that farmers that participated in season-long farmer-led demonstrations in Malawi formed beliefs about the usefulness of the specific CSA practices though these were dependent in part on how similar their own conditions were to the demonstration plot and how well the demonstration plot performed. The authors suggest that the learning process is a two-stage process by which farmers first formulate beliefs based on their own “first-hand and local experience” which then provides an impetus to invest time in learning about the specific practices. These observations seem to indicate a strong link between the ability to recruit higher cognitive networks to learn from observations and execute action subsequently. Thus, this may go beyond the hMNS to include areas of reward and cognitive control as with respect to social learning, reward centers coordinate learning by direct experience (Ramsey et al., 2021).

Learning about sustainable behaviour (e.g., CSA practices) through observation of peers is critical to encourage farmers towards sustainable agricultural practices. This aspect of observational learning and storing the information as part of the brain’s executive functioning, and retrieving the information later to improve future behaviour, supports prospective memory. Successful prospective thinking (described in the next section) enables a person to anticipate a future intention. When evaluating sustainable practices, especially when thinking prospectively, it is important to shift from thinking about individual parts and to adopting a more systems approach by focusing more on the linkages and interactions of each action. Ramsey et al. (2021) have also argued for more research using fNIRS; integrating observational learning with motivations, goals, and intentions and exploring how learning occurs in groups and in real-life situations.

### 4.4. Prospective thinking/memory

Another important factor that determines the likelihood of farmers adopting pro-sustainable behaviour is their ability to project themselves adopting the behaviour in the future, referred to as prospective thinking (Schacter et al., 2012). It requires the ability to flexibly retrieve and recombine information from past experiences into simulation and mental imagery related to future events (Szpunar, 2010; D’Argembeau et al., 2011; D’Argembeau and Demblon, 2012; Schacter et al., 2012). This involves a core network of brain regions, featuring the hippocampus (HC) and the ventromedial prefrontal cortex (vmPFC) (Schacter et al., 2017). The HC plays an important role in recombining memories to mentally simulate future events (Wu et al., 2015). The vmPFC provides contextual details and imagines the future situation (Barron et al., 2013; Benoit et al., 2014).

Brevers et al. (2021) showed that prospective thinking about sustainable behaviours activates a brain network involving the vmPFC, HC, and parahippocampal gyrus. Additionally, activation of vmPFC was triggered during prospective thinking of highly feasible sustainable behaviours. Increasing sustainable behaviours were rated as more feasible suggesting that forming sustainable or “good habits” might be more efficient (Galla et al., 2015; Wood, 2019) or less effortful (Inzlicht and Schmeichel, 2012; Inzlicht et al., 2014) compared with reducing unsustainable or “bad” ones.

Implicit memory interventions have also been suggested that can be further strengthened by neuroscientific tools to monitor processes before behaviour change occurs (e.g., Leeuwis et al., 2022). However, it is argued that only when action is regularly performed does habit emerge which can be defined as automatic responses from memory that led to behaviour in the past (Verplanken and Orbell, 2022). Possible measures have included self-reports of habitised behaviour (Verplanken and Orbell, 2003), reaction time measures of context-response associations (e.g., Neal et al., 2012) or speed of response switching (e.g., Luque et al., 2020).

The following section outlines the most common model used to understand human behaviour. Notwithstanding this, other authors have also highlighted that more research on attitudes, intention behaviour, and habits is warranted (e.g., Gardner et al., 2021).

### 4.5. The theory of planned behaviour

The Theory of Planned Behaviour (TPB) is the most common social-psychological theoretical framework used to understand the dynamics of decision making and human behaviour (Ajzen, 1991; Brosch et al., 2014). It posits that human behaviour is guided by three specific considerations: behavioural beliefs such as the advantages and disadvantages associated with the behaviour; the opinions of significant others towards the behaviour (normative beliefs), and beliefs about possible factors that may hinder or facilitate the performance of the behaviour (control beliefs) (Ajzen, 2019). Moreover, the aggregated beliefs produce a positive or negative attitude, subjective norm (i.e., social pressure to conform to the respective behaviour as a result of normative beliefs), and perceived behavioural control (i.e., to what extent the individual perceives to have control over engaging in the behaviour based on control beliefs) (Ajzen, 2006). These together shape an individual’s behavioural intention. Moreover, the stronger the attitude, subjective norm, and perceived behavioural control the stronger one’s intention is likely to be to perform the behaviour under study (Davis et al., 2002). It is also proposed that an individual will act on their intention where there exists actual behavioural control (perceived behavioural control can act as a proxy) and the opportunity presents itself (Ajzen, 2006). The type of instruments used to measure these constructs are based on elicitation of beliefs in a free-response format (e.g., to understand accessible behavioural beliefs such as advantages and disadvantages of the behaviour which in theory are important determinants of attitude) and then implementation of a questionnaire using self-reports (see e.g., Ajzen, 1991). Extensions to the framework have been proposed such as the incorporation of appraisal-emotion variables which helped to explain additional variance in the intention that is not explained by the current variables (i.e., emotion is only considered as a background factor in the current TPB model) (see Brosch et al., 2014). The authors posit that alongside the TPB variables both the pattern of an individual’s appraisal and an individual’s emotional reactivity in certain situations allow for enhanced understanding of an individual’s intention, especially with respect to engaging in energy-saving behaviours (Brosch et al., 2014). Lalani et al. (2016) found the TPB model explains a high proportion of variation in intention to use CA (a component of CSA) for smallholder farmers in a district of Northern Mozambique. Farmers’ attitude was found to be the strongest predictor of intention followed by perceived behavioural control and subjective norm. More positive environmental beliefs and pro-environmental behaviour have also been linked to brain activity within the DLPFC (e.g., Baumgartner et al., 2019) (Figure 1).

In this section, we have outlined that specific brain activity associated with ST may correlate with other key aspects involved in environmental decision making in this case CSA. Key brain regions/networks of interest include the frontoparietal network extending from the DLPFC to the parietal cortex (PC) a control hub involved in ST and observational learning. Moreover, the network between the DLPFC and nucleus NAc mediates reward processing and motivation which is important for observational learning and pro-environmental behaviour. We posit that it would be important to engage these brain structures to: (a) enhance understanding of CSA practices (e.g., *via* the frontoparietal network) such as tailoring training towards developing ST (e.g., Gray et al., 2019) among farmers and involving observational learning explicitly and (b) motivate farmers to use such practices (*via* the network between the DLPFC and NAc which mediates reward processing and motivation by focussing on a reward/emotion to engage farmers. The following section outlines an interdisciplinary approach to measuring ST, possible correlates of ST, and the use of CSA practices by farmers.

## 5. Proposed theory of change (TOC) and limitations

**Figure 2:**
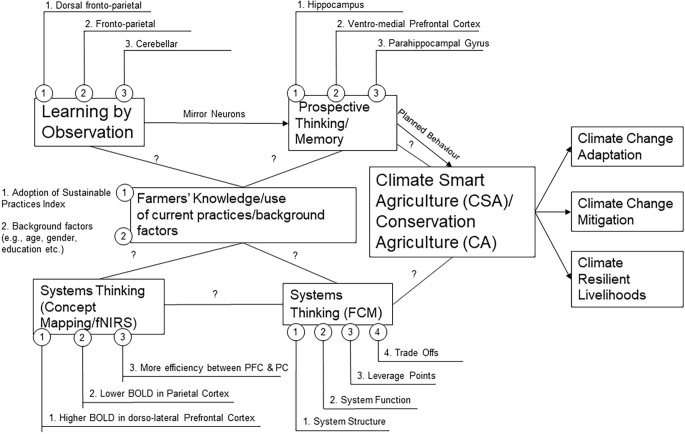
Theory of change. Please note some of the indicators/brain regions highlighted in Figure 2 are based on a scan of the literature and/or the use of fNIRS (e.g., under concept mapping). This might be slightly different with the use of NIRS (focus on the prefrontal cortex).

Figure 2 proposes a Theory of Change (TOC) that will allow us to measure the mechanisms behind systems thinking, correlates with other important aspects of environmental decision making and to what extent this is associated with the use of CSA practices. We have highlighted the possible links/correlations that exist (highlighted by the lines but because the directionality is unknown we have not sought to propose what the effects are in the figure). The numbered nodes from each box/theme highlight possible indicators/metrics that we feel are worth considering. Background factors and the Adoption of Sustainable Practices Index used by Bardenhagen et al. (2020) which provides information on the practices used by farmers and the participation in social learning activities will help to explore to what extent such factors mediate ST/observational learning and use of CSA practices (Figure 2).

Whilst there are clear examples of studies exploring ST with cognitive neuroscience tools (i.e., through concept mapping and the use of fNIRS) in a laboratory setting (e.g., Hu et al., 2019) to our knowledge there is no research to date exploring the relationship between ST and observational learning and/or the use of CSA practices involving mobile neuroscience tools/measures in an LIC. A recent systematic literature review on systems thinking in engineering found that the triangulation of ST *via* multiple assessment types such as the use of concept mapping and fNIRS (Hu and Shealy, 2018) is likely to be beneficial (Dugan et al., 2022). To this end, we have proposed using NIRS and concept mapping similar to the approach taken by Hu and Shealy (2018) and the use of FCM which therefore provides several forms of triangulation (Figure 2).

We propose that higher forms of ST are associated with an enhanced ability to respond to observational learning (e.g., Zonca et al., 2021,^8^) and further posit that this correlates positively with prospective thinking/memory; attitude, subjective norm, perceived behavioural control and thereby intention to perform the behaviour (theory of planned behaviour constructs referred to as planned behaviour in Figure 2). Whilst the observational task designs mentioned in the previous section have utilised fMRI we envisage that it would be possible to adapt methodologies by Burke et al. (2010) and Monfardini et al. (2008), Monfardini et al. (2013) whilst participants are monitored by NIRS and performing tasks using a tablet. For example, observational task designs (e.g., Burke et al., 2010) could be adapted to ask farmers to choose the “best” set of practices to achieve a reward (positive harvest) and avoid a punishment (crop failure) in anticipation of a dry season/drought (with stimuli showing certain CSA practices if employed providing a much higher probability of avoiding crop failure) compared to another set of stimuli with a set of practices less likely to achieve a positive outcome. Another option could be to adapt the approach taken by Monfardini et al. (2008) who compared brain responses in relation to the retrieval of visuomotor associations learned by observation or by trial and error (individual learning). It may also be possible to adapt current visuomotor associations to show farmers a video of a farmer/actor performing a set of agriculture practices (motor responses) and gather post-response feedback (e.g., binary response based on whether the farmer made the “right” choice or not).

Equally, it could be possible to adapt cue-exposure paradigms (e.g., Brevers et al., 2021) to study prospective thinking and context-response associations (e.g., Neal et al., 2012) or speed of response switching (e.g., Luque et al., 2020) for exploration of prospective memory/habit. Cue-exposure paradigms have explored brain activity patterns in response to different cues on “doing more” sustainable behaviours or on “doing less” unsustainable behaviours and the perceived feasibility of performing these practices (Brevers et al., 2021). Likewise, one can imagine a similar cue-exposure paradigm exploring CSA practices and perceived feasibility. For example, more sustainable practices such as minimising soil disturbance, planting a diversity of crops, application of soil cover (“do more”), and unsustainable practices such as crop burning, tillage, and leaving the land bare (“do less”).

Although perceptions of climate change/vulnerability provide useful background factors to include; affective/emotional reactivity is considered an important consideration in providing a better understanding of observational learning in the context of pro-environmental behaviours (e.g., Doell et al., 2021) and the theory of planned behaviour (e.g., Brosch et al., 2014) thus could be incorporated/tested more explicitly using self-reports/questionnaires which can be done in tandem/repeated measurements. We acknowledge the literature on habit (action regularly performed) (e.g., Verplanken and Orbell, 2022) and thus propose a possible feedback from the use of CSA practices to prospective thinking/memory (Figure 2; for a more detailed description of the proposed indicators see Supplementary Table A1, Explanation of key themes and respective indicators).

One of the major challenges in studying environmental decision making more broadly relates to the fact that behavioural changes (e.g., sustainable behaviours/use of certain agriculture practices) are often those which take place in the long-term (Leeuwis et al., 2022). Thus, there are limitations to “one-shot” neuroimaging studies (Sawe, 2017). Whilst the lack of longitudinal is cited as a common limitation of pro-environmental behaviour studies (e.g., Leeuwis et al., 2022), short-term studies may provide a proof of concept such as identifying potential brain regions/networks involved in specific pro-environmental behaviours (e.g., certain agricultural practices) and lead to longer-term studies.

Though some of the studies may be challenging to administer in practice (e.g., observational learning tasks and/or cue/exposure paradigms to study prospective thinking) another option would be to utilise the approach by Baumgartner et al. (2019). The authors use the neural trait approach which explores task-independent, brain-based differences between people and links these differences to a behaviour of interest. The study involved recording task independent EEG at resting before measuring participants’ attitudes regarding environmental behaviour several days later and participants’ everyday pro-environmental behaviour over five days (*via* Smartphone) conducted several weeks later to reduce any carry-over effect (*via* Smartphone). A similar study could be used prior to land preparation and several weeks into the agricultural season, for instance. Similar predictive modelling studies have been done with fNIRS (e.g., see Burns et al., 2018).

Thus, the use of field-based experiments (e.g., Doell et al., 2021) and other trait-based approaches (e.g., Baumgartner et al., 2019) may be more feasible to implement. This would allow for a more nuanced understanding of the indicators that reflect neural and behaviour change at the respective individual level that could support wider population-level studies (Sawe, 2019; Leeuwis et al., 2022). Moreover, nudge theory/choice architecture could also be utilised to investigate the specific “behavioural nudges” that can influence decision making and whether this is associated with higher degrees of ST and exploration of what might be predictive of national behaviour (e.g., Sawe, 2019)^9^. Recent research has found farmers that watched Edutainment TV programmes (e.g., Shamba Shape-up in Kenya) on sustainable agriculture practices had a higher probability of implementing these practices (Areal et al., 2020). The authors concluded that Edutainment TV can effectively “nudge farmers” to implement sustainable agriculture practices and that this highlights a viable approach to addressing challenges such as adaptation/mitigation to climate change (Areal et al., 2020).

## 6. Concluding remarks

The majority of the world’s poorest people reside in rural areas, primarily engaged in agriculture and located in the Global South. Future climate change scenarios have highlighted the negative effects on agricultural productivity worldwide, particularly for LICs in the Global South, highlighting the need for climate change adaptation (e.g., CSA practices) that will contribute to more resilient livelihoods dependent on agriculture. ST has been associated with “better” environmental decision making in a number of environmental and cultural settings, however, to what extent does ST correlate with other important aspects of environmental decision making and improve human adaptive behaviour? Current measures of ST (e.g., cognitive mapping methods such as Fuzzy Cognitive Mapping) are limited in scope (e.g., reliance on recall on participants’ meta-cognition) highlighting the need for triangulation and integration of other approaches to elucidate previously hidden forms of cognition.

Using CSA, as an example case study (with a focus on SSA where the majority of the world’s poorest live^10^) in this perspective essay, we have explored: (i) ST from a social science perspective; (ii) cognitive neuroscience tools that could be used to explore ST abilities in the context of LICs; (iii) an exploration of the possible correlates of systems thinking: observational learning, prospective thinking/memory and the theory of planned behaviour and (iv) a proposed theory of change highlighting the integration of social science frameworks and a cognitive neuroscience perspective that can be used to enhance our understanding of ST and farmers’ decision making. We find, recent advancements in the field of cognitive neuroscience such as Near-Infrared Spectroscopy (NIRS) provide exciting potential to explore previously hidden forms of cognition, especially in a low-income country/field setting; improving our understanding of environmental decision making and the ability to more accurately test more complex hypotheses where access to laboratory studies is severely limited. We posit that it would be important to engage farmers *via* specific brain networks to: (a) enhance understanding of CSA practices (e.g., *via* the frontoparietal network extending from the DLPFC to the parietal cortex (PC) a control hub involved in ST and observational learning) such as tailoring training towards developing ST (e.g., Gray et al., 2019) among farmers and involving observational learning explicitly and (b) motivate farmers to use such practices (*via* the network between the DLPFC and NAc which mediates reward processing and motivation) by focussing on a reward/emotion to engage farmers.^11^

A more nuanced exploration of how contextual factors such as gender and educational efforts such as TV programs might affect these mechanisms would be fruitful. For example, different stimuli (e.g., farmer demonstrations, Farmer Field Schools) combined with different modes of information communication/ social referents) and “behavioural levers” (e.g., nudging) can be important in this regard and warrant further research, particularly from a cognitive neuroscience perspective.

## Data availability statement

The original contributions presented in the study are included in the article/Supplementary materials, further inquiries can be directed to the corresponding author.

## Author contributions

BL, SG, and TM-G conceptualised the study, wrote and approved the final version of the manuscript. All authors contributed to the article and approved the submitted version.

## Footnotes

^1^We have used environmental decision making, human decision making and farmers’ decision making interchangeably. However, there is a large body of literature on farmers’ decision making and other forms of environmental decision making. We have only provided a few examples for the purpose of this perspective piece.

^2^By neural correlates we use the broad definition: “brain activity that ”corresponds with and is necessary to produce a particular experience” (Dingman, 2019).

^3^Whilst this perspective essay has broader relevance for other countries/LICs in the Global South we have mainly focussed on Sub-Saharan Africa and Southern Africa in particular (e.g., Malawi and Mozambique). We envisage piloting the approach in one of these countries where the research team has prior experience of working on this topic and living in the region. Where relevant we have referred to other countries.

^4^For the purposes of this article sustainable agriculture is used interchangeably with CSA. In short, Conservation Agriculture (CA) is defined as the simultaneous application of three principles: (i) minimum soil disturbance (i.e., no-tillage); (ii) maintenance of a permanent soil cover such as through the retention of crop residues, and; (iii) diversification of plant species *via* crop rotations and/or crop sequences/associations. Although context-specific and locally adapted, CA is considered to be the core production system component of CSA which is, however, broader in scope and includes other domains (e.g., De Pinto et al., 2020; for a more detailed description see FAO, 2022a and FAO, 2022b; Mkomwa and Kassam, 2022).

^5^There are many examples, however, where farmers’ indigenous knowledge are climate smart by nature. For example, the indigenous Zai pit system used to improve soil fertility and conserve water in many parts of Africa.

^6^It is beyond the scope of this article to discuss the extensive literature on this topic. For a good overview see Šūmane et al. (2018).

^7^We envisage using NIRS given these specific advantages and its portability/ease of use.

^8^Although Zonca et al. (2021) did not look at ST abilities they found that individuals with a low initial level of strategic sophistication did not succeed in learning from observation compared to those with a higher level of strategic sophistication.

^9^Nudging aims to increase the attractiveness of the behaviour whilst freedom of choice still exists by alteration of the choice architecture (i.e. presenting choices in different ways that will impact decision making) and has been shown to positively influence pro-environmental behaviours (Leeuwis et al., 2022; Mertens et al., 2022).

^10^See World Bank (2022).

^11^Although we have focussed on specific CSA practices we feel that this approach could also be used to explore environmental-decision making in similar/related domains and in different regions/countries.
