# Supplementary material for: Systems Thinking in an era of climate change: Does cognitive neuroscience hold the key to improving environmental decision making? A perspective on Climate-Smart Agriculture
Source: Front Integr Neurosci. 2023 Apr 27;17:1145744. doi: 10.3389/fnint.2023.1145744 (PMC10174047; doi:10.3389/fnint.2023.1145744)
Supplement: Supplementary file 1 [file Table_1.docx]

Table A1. Explanation of key themes and respective indicators

| Theme | Methods/framework | Means of verification | Statistical Analysis | Indicators | References |
| --- | --- | --- | --- | --- | --- |
| Cognitive mapping/Systems Thinking (1) | Fuzzy cognitive mapping | Large scale survey of mental models (fuzzy cognitive mapping) BOLD response data | Hierarchical clustering /Network science metrics and dynamic analysis (scenarios) | Examples include: number of drivers relative to receivers and ordinary variables in the concept map (fewer drivers indicate higher levels of ST), and Simple cycles ratio (e.g. prevalence of feedback loops and thus higher simple cycles ratio indicates higher systems thinking). | Gray et al. (2019), Lalani et al. (2021); Aminpour et al. (2022);Ozesmi and Ozesmi, (2004); Levy et al., 2018). |
| Concept mapping/ Systems Thinking (2) | Concept mapping whilst wearing mobile fNIRS/NIRS* | BOLD response data and scoring concept maps | Graph theory | BOLD response in the prefrontal cortex (PFC). Concept map scores. Network metrics e.g. density (D), global efficiency (E) and clustering coefficient (C) | Hu and Shealy (2018); Hu et al. (2019) |
| Learning by observation (3) | Ecological experience sampling paradigm. Participants report several times per day to what extent they had recently performed or were exposed to by others (either first-hand or learned about) environmentally relevant behaviours (ERBs) as well as their current affective state (Doell et al., 2021). | Field experiments and self-reports/questionnaire administered by Smartphone. Other scales/questions if relevant feasible used in Doell et al. (2021). For example, Environmental Trait Affect Questionnaire.(taken pre-test and monitored over duration of study to investigate relationship with ERBs)^.^ | Multilevel binomial logistic regression model/Multilevel linear regression | Frequency of Positive ERBs and negative ERBs. Number of ERBs exposed to by others (either first-hand or learned about). | Doell et al., (2021) |
| Prospective thinking/memory (4) | Cue-exposure paradigms | Specific cue-exposure design can be designed for CSA practices. For example, more sustainable practices such as minimising soil disturbance, planting a diversity of crops, application of soil cover (“do more”) and unsustainable practices such as crop burning, tillage, leaving land bare (“do less”). Feasibility scale. | General linear model (GLM) | Regions that exhibit greater activation for the ‘do less’ cue in the ‘do less minus do more’** rating scale of how feasible it is to implement sustainable/unsustainable practices | Brevers et al. (2021) |
| Planned behaviour (5) | Theory of Planned Behaviour/extended model | Survey and Key Informant interviews/Focus group discussions | OLS/hierarchical regression | Attitude, Subjective Norm, Perceived Behavioural Control and Intention/related beliefs. | Examples: Brosch et al., (2014); Lalani et al., 2016) |
| Use of practices/on-farm measurements (6) | On-farm measurements; farm budget | Verify existence of CSA practices on farmers’ main plot through field visits; household survey; Soil sampling and measurements of other metrics as required e.g. biomass/yield etc. | Simple T tests of ‘experimental’ treatments to ‘control’ treatments | Number and type of practice use (visual inspection). Biomass/yield, weed cover, and soil quality indicators. Gross margin per hectare. Other household indicators where feasible/relevant | See Lalani et al. (2021) (e.g. FCM was used to elicit representations of farmers’ mental models for 50 farmers and on-farm measurements were taken from their main plot of land.) |

*This may be time-consuming to administer at a large-scale ** This will need to be thought through to see what would be feasible with use of NIRS (limited spatial resolution) compared to fMRI used in the Brevers et al. (2021) study.

Description and limitations:

We envisage that there would likely be several phases of data collection. Phase 1 would require eliciting information (e.g. Focus group discussions) to explore farmers’ views on their agricultural system/practices and beliefs related to theory of planed behaviour. Phase 2 will require construction of questionnaires which can be administered at a large-scale (e.g. randomised sampling of farmers in a specific region/appropriate sampling methodology). This will enable construction of cognitive maps (FCM) and concept maps and gathering of brain activity related data (BOLD response) via the use of NIRS. To what extent concept mapping can be combined with the use of NIRS will require further exploration/a rigorous pilot study and support of a neuroscientist. Phase 3 will aim to sample/follow a subset of farmers from Phase 2. Similarly, adapting the approach used by Doell et al. (2021) would enable exploration of observational learning and pro-environmental behaviour. We also propose that it may be possible to adapt cue-exposure paradigms either to explore prospective thinking/memory such as adapting those used by Brevers et al. (2021) and/or as another method to explore observational learning such as adapting the learning by observation task (LeO) (e.g. Mofardini et al., 2013). With respect to the theory of planned behaviour constructs (5) time-constraints may require utilisation of a proxy and in this case adapting the pro-environmental behaviour questions used by Doell et al. (2021) may be possible. Similarly, it may be time consuming/difficult to administer cue-paradigm tasks at a large-scale and an alternative is to measure self-reports over time via smartphone as used by Doell et al. (2021). Background factors/adoption of sustainable index (e.g. knowledge/use of practices and engagement in social learning activities) farm budget/verification of current practices can be elicited from household survey and this can be incorporated into phase 2. Phase 4 (if feasible) will include gathering relevant on-farm measurements (e.g. Lalani et al. 2021).

Statistical analysis/merging domains:

Specific statistical analysis/how to merge the different components will require further exploration/teasing out. The key indicators of ST can be used as predictor variables alongside exposed to ERBs (learning by observation), BOLD response (cognitive activation) etc. and/or dummy variables can be created where needed (e.g. 1= higher forms of ST; 0=lower forms of ST). With respect to the theory of planned behaviour variables commonly included as background factors include age, gender, education, intelligence, knowledge, past behaviour, personality traits etc. (Ajzen, 2019). Therefore, it may be possible to include the various variables as background factors in the TPB model/regression analysis.

As explained in the proposed TOC section a similar approach to the one used by Doell. et al (2021) could be used. For example, Baumgartner et al. (2019) use a neural-trait approach to explore whether baseline activation in the right lateral Prefrontal cortex (PFC) explains additional variance in pro-environmental behaviours which cannot be explained by general attitudes towards the environment. They used the New Environmental Paradigm (NEP) which is the most widely used measure of environmental concern consisting of 15 items about environmental views. (these items could thus be used as an alternative to some of the theory of planned behaviour constructs if it is deemed too time consuming to administer). (Dunlap et al., 2008) To this end, Baumgartner et al. (2019) in their model employed a hierarchical regression with pro-environmental behaviour scores as the dependent variable, and NEP score and delta current density in the right lateral PFC as first and second predictor, respectively. The authors found that NEP score correlated with daily pro-environmental behaviour on a trend level (p=0.09) and including an additional predictor (delta current density in the right lateral PFC) in the regression explained additional variance. We could thus adopt a similar approach adapting the pro-environmental behaviour measures to account for observational learning (e.g. ERB measures used by Doell et al., 2021) which can be used as the dependent variable and using the NEP scores (environmental attitudes) and similar metric to delta current density in the right lateral PFC (one measured by NIRS e.g. BOLD response) as a predictor. Moreover, we could also include a dummy for systems thinking as suggested alongside other predictors/background factors (e.g. age, education, gender etc.).
